# Effects of Olfactory and Auditory Enrichment on Heart Rate Variability in Shelter Dogs

**DOI:** 10.3390/ani10081385

**Published:** 2020-08-10

**Authors:** Veronica Amaya, Mandy B.A. Paterson, Kris Descovich, Clive J.C. Phillips

**Affiliations:** 1 Centre for Animal Welfare and Ethics, School of Veterinary Sciences, University of Queensland, White House Building (8134), Gatton Campus, Gatton, QLD 4343, Australia; mpaterson@rspcaqld.org.au (M.B.A.P.); k.descovich1@uq.edu.au (K.D.); c.phillips@uq.edu.au (C.J.C.P.); 2 Royal Society for the Prevention of Cruelty to Animals, Queensland, Wacol, QLD 4076, Australia

**Keywords:** dog, heart rate variability, shelter, stress, arousal, lavender, dog appeasing pheromone (DAP), music

## Abstract

**Simple Summary:**

Many pet dogs end up in shelters, and the unpredictable and overstimulating environment can lead to high arousal and stress levels. This may manifest in behavioural problems, and decreased welfare and adoption chances. Heart rate variability is a non-invasive method to measure autonomic nervous system activity, which plays an important role in the stress response. The sympathetic nervous system is responsible for increasing the dog’s arousal in response to stress and the parasympathetic nervous system is responsible for counteracting the arousal and calming the dog. Environmental enrichment can help dogs to be more relaxed, which is likely to be reflected by increased parasympathetic activity. Dogs’ heart rate variability responses to three enrichment methods capable of reducing stress—music, lavender and a calming pheromone produced by dogs, dog appeasing pheromone and a control condition (no stimuli applied) were compared. Exposure to music appeared to activate both branches of the autonomic nervous system, as dogs in that group had higher heart rate variability parameters reflecting both parasympathetic and sympathetic activity compared to the lavender and control groups. We conclude that music may be a useful type of enrichment to relieve both the stress and boredom in shelter environments.

**Abstract:**

Animal shelters can be stressful environments and time in care may affect individual dogs in negative ways, so it is important to try to reduce stress and arousal levels to improve welfare and chance of adoption. A key element of the stress response is the activation of the autonomic nervous system (ANS), and a non-invasive tool to measure this activity is heart rate variability (HRV). Physiologically, stress and arousal result in the production of corticosteroids, increased heart rate and decreased HRV. Environmental enrichment can help to reduce arousal related behaviours in dogs and this study focused on sensory environmental enrichment using olfactory and auditory stimuli with shelter dogs. The aim was to determine if these stimuli have a physiological effect on dogs and if this could be detected through HRV. Sixty dogs were allocated to one of three stimuli groups: lavender, dog appeasing pheromone and music or a control group, and usable heart rate variability data were obtained from 34 dogs. Stimuli were applied for 3 h a day on five consecutive days, with HRV recorded for 4 h (treatment period + 1 h post-treatment) on the 5th and last day of exposure to the stimuli by a Polar^®^ heart rate monitor attached to the dog’s chest. HRV results suggest that music activates both branches of the ANS, which may be useful to relieve both the stress and boredom in shelter environments.

## 1. Introduction

Animal shelters are stressful environments due to novelty, loud noises, unpredictability and lack of control [1,2]. This overstimulating environment can lead to high arousal levels and stress in shelter animals. The stress response is multifactorial and includes activation of the hypothalamic–pituitary–adrenal (HPA) axis and the sympathetic branch of the autonomic nervous system (ANS), with behavioural [3] and physiological changes [4] produced. Behavioural responses to stress consist of increased arousal [5,6], which in turn results in heightened sensory sensitivity and alertness, the production of corticosteroids and increased heart rate (HR) [7]. Stress in animals can be monitored in various ways, such as behavioural observation, which provides external indicators of an animal’s internal state [8] and the response to its surroundings, physiological measures such as the amount of circulating glucocorticoids [9] and heart rate variability (HRV) [10]. HRV is a useful indicator of ANS activity [11] and has the advantage of being measured non-invasively [10,12] (externally and without puncturing the skin).

The ANS is divided into two branches: the sympathetic nervous system (SNS), which is excitatory, and the parasympathetic nervous system (PNS or vagal), which is inhibitory [13]. When there are no apparent threats, the PNS is dominant, which helps to maintain low levels of arousal and a stable heart rate [13]. PNS activity is mediated by acetylcholine neurotransmission released by the vagus nerve [14] and it produces a rapid response in cardiovascular function [15]. The SNS becomes dominant in situations of psychological or physical stress, leading to arousal that helps the individual to respond to environmental challenges [13]. SNS activity is mediated by epinephrine and norepinephrine [14], producing changes in cardiovascular function in a slower time course than PNS [15].

HRV is the fluctuation of time intervals between successive heart beats [16] and reflects the interaction between both branches of the ANS on the sinoatrial node of the heart [10]. A healthy heart has irregular time intervals between beats [17,18], therefore a high variability in sinus rhythm suggests better health and cardiovascular adaptability [19]. Low variability can indicate abnormal cardiac activity or an ANS imbalance leading to poor adaptability to psychological and physiological challenges [19]. HRV is assessed through several time domains, frequency domains and non-linear parameters (Table 1).

HRV can be recorded using standard electrocardiogram (ECG) equipment, such as Holter systems, and wearable devices such as Polar^®^ heart rate monitors [11]. The recorded RR intervals (duration between two consecutive R waves of the ECG) are then analysed using HRV software such as Kubios. The measurement of HRV can be challenging in terms of accuracy and interpretation. One key challenge is determining whether all traces are valid or if some are artefacts. Artefacts are recordings that appear like heartbeats but are not produced by sinoatrial node depolarisations and therefore are abnormal [16]. They can occur due to technical factors, such as poorly placed or fastened electrodes, movement of the subject and/or long recordings [25,26,27]. Artefacts can also occur due to physiological factors, such as ectopic beats, ventricular tachycardia and atrial fibrillation [25,26]. Data should be corrected for artefacts [11], as otherwise they can affect the reliability of the results [25,27]. HRV is influenced by many factors, such as respiration, posture and physical activity, and therefore the conditions under which data are collected should be standardised (i.e., stationary subject) [11] to allow treatment effects to be identified.

Studies in cattle (*Bos taurus*) [28], horses (*Equus caballus*) [20] and dogs (*Canis familiaris*) [29] have investigated the association between stress and HRV. In calves, root mean square of differences between the successive RR interval (RMSSD) was significantly reduced in those with external stress load (ambient temperature >20 °C and insect disturbance) and internal stress load (diarrhoea) compared to animals without obvious stress load. Standard deviation of RR intervals (SDNN) was significantly reduced in calves with internal stress load compared to those experiencing external stress load or no evident stress load [28]. In horses, there was a significant increase in HR, low frequency (LF) and the LF/HF ratio and a significant decrease in HF when they were forced to move backwards for 3 min compared when at rest or when forward walking [20]. In dogs approached by a stranger in the absence of their owner, HR increased and SDNN decreased [29]. Maros et al. [30] found that when dogs looked at their favourite toy, SDNN significantly increased, possibly indicating elevated attention. Kuhne et al. [31] found that when dogs had increased HR and decreased RMSSD compared to baseline values, they performed more appeasing and redirected behaviours. Moreover, low percentage of successive RR interval pairs that differ by more than 50 ms (pNN50) has been associated with aggression; this parameter was significantly lower in dogs with bite histories compared to dogs without them [32]. These results indicate that HRV is a useful tool to assess physiological and emotional stress.

As mentioned above, shelters can be challenging places for dogs and it is important to try to mitigate stress and arousal levels to avoid chronic stress that may impact welfare [33]. Moreover, stress and high arousal levels can increase the development of undesirable behaviours [3,34,35,36]. These reduce the likelihood of adoption [37], increase the risk of being returned to the shelter after adoption [38,39] and elevate the risk of euthanasia [2]. Sensory environmental enrichment, which consists of stimulating one or more of an animal’s senses [40] is a useful tool to help reduce stress and arousal levels.

In humans, music has been effective in reducing anxiety in patients during hospitalisation [41], and can enhance relaxation by masking unpleasant noises [42]. Music can also reduce anxiety in patients with coronary heart disease, cancer patients and those awaiting surgery [43]. Music has been effectively used in animal studies as a form of environmental enrichment, for example, Western lowland gorillas (*Gorilla gorilla gorilla*) tended to perform more behaviours suggestive of relaxation when exposed to classical music compared to a no auditory stimulation control [44]. Additionally, Asian elephants *(Elephas maximus)* showed less stereotyped behaviours when exposed to classical music compared to a no auditory stimulation control [45]. Kennelled dogs have been experimentally exposed to different types of auditory enrichment. Kogan et al. [46] examined the effects of different types of music and found that with classical music dogs spent more time sleeping and less time barking than with heavy metal, bespoke music specifically designed for dog relaxation, or no music. Bowman et al. [47] used a variety of music genres (Soft Rock, Motown, Pop, Reggae and Classical) and found that when any type of music was played, dogs spent less time standing and more time lying (with the exception of Reggae). Wells et al. [48] played different types of music (Classical, Heavy Metal and Pop), as well as human conversation, and found that dogs exposed to classical music spent more time resting and less time standing than dogs exposed to the other treatments. In Bowman et al. [9], the initial effects of classical music compared to a silent control, were a reduction in vocalisation and increase in time lying down, but dogs habituated to the stimuli by the second day of exposure.

Lavender exposure has been associated with increased relaxation [49] and reduced anxiety in humans [50,51], and has also been shown to have beneficial effects in different animal species. In pigs (*Sus scrofa domesticus*), lavender straw appeared to reduce the severity of travel sickness [52]. Horses exposed to humidified air mixed with lavender essential oil had lower heart rates, after an acute stress response, than horses exposed to humidified air alone [53]. In mice (*Mus musculus*), lavender was shown to have a sedative effect after inhalation, reflected by decreased motility of the animals [54,55]. Similarly, lavender has been used in dogs in different environments. Graham et al. [56] used diffused essential oils in a rescue shelter, and found that dogs spent more time lying down and less time moving when exposed to lavender and chamomile oils compared to rosemary and peppermint oil and a control (no odour). Wells [57] studied the effects of lavender for travel-induced excitement in dogs. Dogs were exposed to a lavender impregnated cloth and a control cloth (no odour) while going on car journeys. Dogs exposed to lavender spent more time resting and less time vocalising and moving. A study in sheep *(Ovis aries)* showed that lavender effects depended on the sheep temperament. Calm sheep exposed to lavender oil had a lower agitation score and vocalised less than calm control sheep, while nervous sheep exposed to lavender vocalised and attempted to escape more than nervous control sheep [58].

Dog appeasing pheromone (DAP) is a synthetic compound based on fatty acids secreted by the mammary gland of bitches after parturition [59]. The effect of the DAP diffuser use has been studied in puppies with disturbance (i.e., vocalisation and continuous door scratching at night) and house-soiling issues. Puppies exposed to DAP cried significantly less than those exposed to a placebo, but there were no effects on the number of nights that puppies soiled inside [60]. Dogs using impregnated DAP collars showed some improvement in behaviour while in car journeys. The greatest improvement was in dogs that had shown motion sickness signs (vomiting and salivating), while the least was in excitable dogs (those who had shown behaviours as barking, jumping and whining) [61]. In a veterinary clinic setting, DAP diffusers appeared to reduce anxiety signs, but there was no evidence of aggression reduction during a clinical exam with a single exposure to the pheromone [62]. In shelter dogs, DAP diffused for 7 days reduced barking amplitude and frequency when people walked by the kennels [63]. DAP collars have been used in puppies during training sessions where they appear to result in less fearful and more sociable behaviour, and improved learning [64]. This literature shows that sensory environmental enrichment can help to reduce stress and arousal signs in different settings and different animal species.

This study is part of a larger project investigating enrichment effects in shelter dogs (methodology and behaviour results are reported in Amaya et al. [65]). The first part of this project analysed the behaviour of sixty dogs when exposed to music, DAP, lavender or a control [65]. Dogs performed fewer vocalisations and increased calmer body postures when exposed to any of the treatments compared to the control, although the effect was weaker for the lavender treatment [65].

The current paper reports on the physiological data collected from the shelter dogs during the study described in Amaya et al. [65] and the aim was to determine if the stimuli have physiological effects that are detectable through HRV recordings. We hypothesized that HRV parameters influenced by vagal activity will be higher in shelter dogs exposed to music, lavender and DAP than those in the control group.

## 2. Materials and Methods

### 2.1. Subjects

The subjects enrolled in the study consisted of 60 shelter dogs; 35 males and 25 females, all desexed. Mean (± SD) dog age was 3.2 ± 2.4 years, ranging from 6 months to 11 years. They came from different sources and most were mixed breed. Their mean length of stay in the shelter was 45.9 ± 29.8 days, range 8–150 days (Appendix A). On admission to the shelter, all dogs were given a veterinary clinical examination and a standardised behaviour assessment as described in Clay et al. [66]. Each week, the RSPCA Qld Behaviour Team identified dogs for the study, with inclusion criteria being those showing high arousal-related behaviours, such as air snapping, mouthing, attempts to bite their lead or handler, excessive activity, constant vocalisation and over-reaction to other dogs. The selection was made based on information of their kennel behaviour, as provided by shelter staff working with them regularly. Shelter staff were responsible for placing the selected dogs into the study kennels; they were blind to the treatments and assigned dogs at random to each kennel as they became available.

### 2.2. Study Environment

This study was conducted at the Royal Society for the Prevention of Cruelty to Animals Queensland’s (RSPCA Qld) Animal Care Campus at Wacol, Brisbane, Australia, between August and November 2017. Shelter activities took place as usual (cleaning, feeding and walking) and therefore shelter staff and volunteers were regularly present around the kennel blocks. Two kennel blocks were used for this study, each consisting of 16 kennels divided into two rooms of 8 kennels (two rows of four) and separated by a door. Each kennel had dimensions of 1.6 m × 4 m, and included a crate measuring 0.72 m × 1.55 m and a bed. Both sides had plastic walls that prevented dogs from seeing each other. The back of the kennel had thin metallic bars from roof to floor and the front door had a solid section at the bottom and the same metallic bars from the top of the solid section to the top of the door. For housing details refer to Amaya et al. [65]. The dogs were fed dry food twice a day and had water ad libitum. They were taken for walks twice a day by volunteers for 10 min each time (during the morning cleaning and the afternoon spot cleaning) and had occasional contact with volunteers at other times, except for the 3 h treatment period and 1 h post-treatment.

### 2.3. Study Design

Dogs were exposed to one of three forms of enrichment: music (*n* = 14), lavender (*n* = 15) and DAP (*n* = 16) or a control condition (no stimuli applied; *n* = 15). Dogs were exposed to the stimuli in their kennel for 3 h/day on 5 consecutive days, but the HRV measurement only took place on the final day of exposure to the stimuli. Treatments were conducted between approximately 10.30 am and 13.30 pm, depending on when all morning activities were complete. Dogs were also monitored for one-hour post-treatment.

For the music treatment, a databank of 301 songs was downloaded from Spotify (www.spotify.com/au/), and filters were applied to these songs to select music believed to be most suitable for the dogs. Songs were included if they matched the following criteria: tempo of 70 or fewer beats per minute, valence from 0 to 0.5 and energy less than 0.2 (these two last on scales of 0–1.0) [67]. The piano was the sole instrument, except in 6 tracks in which there was accompaniment by violins for part of the tracks [65]. Previous research suggests that single instruments require less neurological processing than multiple instruments [68]. The resulting selection of 51 tracks with a total 183-min duration was played with random track selection order on a mobile phone (Motorola^®^ mobility (Google), Moto G (1st generation), Mountain View, CA, USA) connected to a mobile wireless stereo speaker (Logitech^®^, X300, Lausanne, Switzerland), with a set volume throughout the experiment. The speaker was placed in a plastic holder hung on the crate’s door (in the middle of the kennel). The music was played at 70 dBA, measured from the kennel’s door (700 cm of distance from speaker) using a sound level meter (Digitech^®^, QM-1589, Stanford, CT, USA) at the beginning of each treatment period.

For the lavender treatment, one ultrasonic diffuser (Select Botanicals, Gladesville, New South Wales, Australia) was placed in the crate and another at the back of the kennel. The dilution was 4 drops of 100% organic Bulgarian lavender (*Lavandula angustifolia*; Select Botanicals, Gladesville, New South Wales, Australia) in 60 mL of water. For the DAP treatment, 3 pumps of a synthetic analogue of the canine appeasing pheromone (15.72 mg/mL; Adaptil^®^, Ceva, Glenorie, New South Wales, Australia) were sprayed on a bandana worn by the dog and 2 pumps on the dog’s bedding as recommended by the manufacturer. Three additional pumps were sprayed at different points of the kennel’s floor (1 at each of the back corners and 1 the front door). The control dogs did not receive any extra sensory stimulus.

### 2.4. Data Collection and Analysis

On the 5th day of every research week, the dogs were fitted with a heart rate monitor. Four human heart rate monitors were used throughout the study, randomly allocated to treatments. Two different models were used: 3 Polar^®^ RS800CX (Polar Elctro, Kempele, Finland) and 1 Polar^®^ V800 (Polar Elctro, Kempele, Finland). They consisted of a wearlink strap, a watch-computer and a wireless integrated network device. The Polar^®^ RS800CX has been validated for measuring heart rate variability of dogs [69,70,71,72] and employed in studies using music as environmental enrichment [9,47,73]. The Polar^®^ V800 has been validated for measuring heart rate variability in humans [74].

The heart rate monitor was positioned on the left side of the thorax at the third intercostal space and secured with adhesive bandages (ZebraVet^®^, Rocklea, Queensland, Australia). The area of attachment was shaved and cleaned with methylated spirits to allow good contact between the device and the skin. Ultrasound liquid was generously applied to the device to help with the transmission. The watch-computer was secured to the dog’s collar. The heart rate monitor recorded for 20 min before commencing the treatment to capture a baseline of the heart rate. It then recorded for three hours while the dog was being exposed to the treatment. An extra hour was recorded after the treatments had finished to measure after-effects. Every 45 min the watches were checked to make sure they were still recording. If they had stopped, more ultrasound liquid was added and the recording restarted. The procedure for the heart rate monitor positioning and securing with adhesive bandages was the same for dogs in the four treatments.

Once the recording finished, data were transmitted via a bidirectional infrared interface to the Polar^®^ Protrainer 5 software (Polar Electro, Kempele, Finland) for the Polar^®^ RS800CX and via USB connection to the Polar^®^ FlowSync software (Polar Electro, Kempele, Finland) for the Polar^®^ V800. These data were then exported as text files to Kubios software (Standard Version 3.1.0. Kubios Oy (limited company) Departments of applied Physics, University of Eastern Finland, Kuopio, Finland).

Dogs were video recorded in their kennels using two mini cameras with charge-coupled devices and infrared capability (Signet^®^, Electus Distribution Pty. Ltd., Rydalmere, New South Wales, Australia), one at the front and one at the back of the kennel. Behaviours were recorded continuously (24 h/d during the 5 d of stimuli exposure). Observations were divided in three periods: the treatment period (3 h) 5 min observed every 15 min, i.e., 12 separate observations lasting 3600 s in total; the post-treatment period (4 h), 5 min observations every 30 min, i.e., 8 separate observations lasting 2400 s in total and the night period, 5 min of each hour were observed, i.e., 16 separate observations lasting 4800 s in total. Boris^®^ behaviour coding software (version 6.0.4. for Windows, Torino, Italy) was used to record behaviour in an ethogram [65]. There was a single coder for all the videos and they were not blind to the stimuli as specific objects of each treatment were visible in the videos (i.e., bandana, speaker and diffusers). Time values were then transformed into % values (duration of behaviour/total observation time × 100, in s). Videos were observed for a second time during the baseline, treatment and post-treatment periods of the 5th and last day of exposure to the stimuli, when HRV was recorded, to find segments where the dogs were lying down for 5 consecutive minutes. This position was chosen as movement can interfere with the recordings and create artefacts. It has been recommended to obtain HRV during conditions when the subject is stationary, with unchanging motor activity [11,14]. As the dogs were freely moving in the kennel, the only possible segments of 5 consecutive minutes in the same position were obtained when the dogs were lying down. Five 5-min segments that fit the position criteria and had the smallest percentage artefact correction were chosen for each dog during the treatment period; this segment length has been recommended to standardise HRV studies [11,14]. Data were analysed either uncorrected or corrected using the ‘very low threshold’ option of this software (0.45 s) and only segments with less than 5% corrected beats were included in the analysis following Kubios [75] recommendations. Of the 60 dogs originally enrolled in the study, 5 were excluded from the HRV analysis for the uncorrected data analysis, and 26 from the corrected data analysis. One dog was adopted the day before the HRV analysis took place. For the corrected data, the excluded dogs either did not fit the requirement of having segments with less than 5% artefact correction while lying down (*n* = 17) or did not meet the criteria mentioned above and also had missing data due to technical issues with the Polar^®^ straps and/or watches (*n* = 8). No attempt was made to interpolate data for missing dogs. Therefore, data from 55 and 34 dogs were included in the uncorrected and corrected HRV analysis of treatment effects, respectively: music (*n* = 12 and 6), lavender (*n* = 13 and 10) and DAP (*n* = 16 and 9) or the control condition (*n* = 14 and 9). Baseline and post-treatment data were not used as only 14 and 7 dogs, respectively, had segments fitting the standard requirements. Baseline values would have been a useful measure as the dog’s own control, but it was only recorded for 20 min and therefore it was hard to find 5 min segments when dogs were lying down and furthermore, with less than 5% artefact correction. Due to the large imbalance in dog numbers between treatments, the baseline data could not be statistically analysed.

### 2.5. Statistical Analysis

The HRV data were statistically analysed using Minitab 18 software (Minitab. LLC, State College, PA, USA). Mixed effects models were constructed using dog and heart rate monitor (HRM) as random factors, with dog nested within HRM, and treatment as a fixed factor. Dependent variables were Mean RR (ms), Mean HR (bpm), SDNN (ms), RMSSD (ms), pNN50 (%), standard deviation 1 of the Poincare Plot—short-term HRV (SD1), standard deviation 2 of the Poincare Plot—long-term HRV (SD2), LF/HF ratio, LF band (0.067–0.235 Hz) and HF band (0.235–0.877 Hz). These bands were estimated specifically for dogs by Behar et al. [22]. Both bands are expressed in absolute values of power (ms^2^) and normalised units (n.u.). Artefacts were also fit as a dependent variable.

Assumptions were checked via plotting, and square root transformations were used for absolute LF power (ms^2^), absolute HF power (ms^2^) and LF/HF ratio, to achieve normal distribution of residuals. Assumptions were met after transformation. R-squared values for all models were high (>68% for HRV parameters and 65% for artefacts). When omnibus tests were significant (*p* < 0.05), differences between individual treatments were examined using Tukey’s tests, which adjust for multiple comparisons. Trends were considered if *p* ≤ 0.10 but >0.05.

In the first study from this project [65], treatment effects on the behaviour of dogs (*n* = 60) were analysed using mixed effects models constructed using dog as a random factor and dog number (entry time to the study), treatment and day as fixed factors. Only a subset (*n* = 34) of the dogs from that study were able to be included in the current dataset, therefore to assist in the interpretion of HRV treatment effects, the behaviour was reanalysed using the same statistical model but only using results from dogs with both behaviour and HRV data.

## 3. Results

### 3.1. Artefact Correction and Model Selection

There was no significant treatment effect on artefact correction (F_3,26_ = 0.75, *p* = 0.53) with mean correction levels of 1.88%, 1.42%, 1.42% and 2.23% (SED = 0.49) for music, lavender, DAP and control treatments, respectively. We selected the model that used artefact correction because that method is generally recommended by those working in this field [11,25,27]. R-squared values were high (>70%) for both uncorrected and corrected models.

### 3.2. Treatment Effects on HRV during the 3 h Treatment Period

Absolute LF power (ms^2^) was higher in dogs exposed to music compared to those in the lavender and control groups (Table 2). SD2 (ms) was higher in dogs exposed to music compared to those in the lavender group. There were trends for treatment effects on mean HR, mean RR (ms) and SDNN (ms) (*p* = 0.10, 0.08 and 0.07, respectively). Inspection of the means suggest that these trends are largely influenced by the music group, which had the lowest mean HR, and highest mean RR/SDNN of the treatment/control groups. There were no significant treatment effects for any of the other HRV parameters.

### 3.3. Treatment Effects on Behaviour of Subset of Dogs (n = 34) during the 3 h Treatment Period

Reanalysis of the behaviour data from the previous study with the current animal cohort resulted in some differences to the statistical outcomes (Table 3 and Appendix B). For 11 of the 20 behaviours analysed, the deduced statistical significance (significant: *p* < 0.05, trend: 0.05 < *p ≤* 0.10, or non-significant: *p* > 0.10) was the same. Two behaviours were no longer significant in the subset cohort (lie down total and sniff ground) and two lost significance and became trends (lie down-head down and body shake; Table 3) Two behaviours were no longer trends (stand and walk) and two became trends (groom and tail still). One behaviour reached criterion for significance in the subset cohort but did not in the full behavioural study (lie down-head up). It is important to note that while the HRV data was only recorded on the 5th and last day of exposure to the stimuli and only segments when dogs were lying down were analysed, the behaviour data belongs to the 5 days of treatment exposure (3 h/d) and therefore includes all the observed behaviours (i.e., standing).

In the subset of dogs, those in the DAP group laydown with their head up more than dogs in the music or lavender groups. Dogs in the control group stood more on their hind legs with their front legs on the exit door and vocalised more than dogs in the three stimuli groups. Dogs in the music group panted and wagged their tail less than those in the control group. There was a trend for treatment effects on lie down-head down. Inspection of the means suggest that these trends are influenced by the music group, which spent the most time lying down with the head down out of the treatment/control groups. In both datasets, dogs exposed to the stimuli showed more behaviours related to relaxation compared to the control group, but in the full dataset the lavender group did to a lesser extent compared to the other two stimuli.

## 4. Discussion

### 4.1. Stimuli Effects on HRV Parameters

Dogs from the music group had a higher absolute LF power (ms^2^) than dogs in the lavender and control groups. The interpretation of the LF band has been debated in the literature. Some studies [76,77,78,79] consider it an index of sympathetic activity only, while others suggest that this band reflects a mix of parasympathetic and sympathetic activity [23,80,81,82,83,84,85,86,87,88,89,90]. This second argument is based on research that shows conditions associated with sympathetic activity and therefore an increase in LF power would be expected, but instead a decrease in LF power has been observed [14], for example, during myocardial ischemia [80] and exercise [80,91,92]. Moreover, pharmacological interventions to enhance or reduce sympathetic activity in the heart do not produce consistent changes in the LF power [90,91,93]. Based on this, we have interpreted the LF band as a parameter that is influenced by both parasympathetic and sympathetic activity.

A relationship between the LF band and music has been previously established in humans. It was found that the LF component increased with the number of music sessions people were exposed to, for both calming and excitative music, and decreased when no music was played [94]. It was concluded that the LF component increases with music listening regardless of music type, and musical stimuli might activate both parasympathetic and sympathetic nervous systems as even brief exposure to music can produce perceptible cardiovascular effects [95] and the beat of music alone can cause a response in the ANS [43]. Yet, both calming music and silence produced subjectively relaxing moods [94]. These results concur with our findings, as the absolute LF power (ms^2^) of the music group was higher than for lavender and control, two non-auditory conditions. This suggests that this parameter reflects the presence of musical stimuli. This possibly activated both branches of the ANS due to its varied effects temporally, with different rhythms and cadences in the tracks used.

The first study of this project compared the effects of the three stimuli and a control condition on behaviour [65]. Although the HRV results suggest that music activates both branches of the ANS, dogs from that group spent significantly more time lying down with their head down and less time standing on their hind legs with their front legs resting against the exit, vocalising and panting compared to the control group (Appendix B). These results were not identical when behaviour analysis was run in the subset of dogs drawn from the first study for the present analysis. However, in both analyses dogs in the music group stood on their hind legs with their front legs resting against the exit, vocalised and panted less than the control group. In this subset of dogs there was also a trend for a treatment effect on lying down with the head down, which appeared to be highest in the music group (Table 3). All of these behaviours are associated with increased relaxation, which corresponds with the data in the previous study using the full cohort of dogs.

SD2 is a non-linear parameter that describes long-term variability and is correlated with SDNN and RMSSD [96]. It is influenced by both sympathetic and parasympathetic input [24,96,97,98,99]. Tulppo et al. [99] found that SD2 decreased during exercise after parasympathetic blockade and therefore attributed it to sympathetic activation. Consequently, an increase in this parameter is thought to indicate a decrease in sympathetic activity. Previous studies have found higher SD2 in dogs exposed to classical music compared to a silence control [9,73]. Bowman et al. [9] interpreted it as a decrease in sympathetic activity, associated to decreased anxiety in the dogs. In our study, dogs in the music group had a higher SD2 compared to lavender. As mentioned above, dogs in the music group in this subset of dogs had a trend to lie down with their head down more than the other three groups. Moreover, the lavender group showed behaviours associated with increased relaxation and reduced arousal compared to the control group to a lesser extent than the music and DAP groups in the full dataset analysis. This suggests that the difference in SD2 could be due to lower sympathetic activity in the music group or increased sympathetic activity in the lavender group. However, the lack of difference in other parameters specific to vagal activity makes any firm conclusions difficult.

In humans, several studies have tested the effects of lavender on HRV and other cardiac parameters (i.e., heart rate, systolic and diastolic blood pressure), with no significant effects [100]. However, a dog study had some significant findings. Dogs received either a dermal application of lavender or a placebo during four 3.5 h periods while monitoring HRV. In dogs exposed to lavender, there was a significant increase in HF power and a significant decrease in heart rate, but only during the 3rd and 4th periods, respectively [101]. These results suggest that topical exposure to lavender oil had some effect on vagal activity. The difference in results with our study might be due to the fact that lavender was administered through diffusers rather than on the skin, which may decrease any anxiolytic effect. Further research would be recommended on the effect of application method.

There was a trend for dogs in the music group to have lower mean HR and higher mean RR, which reflects increased vagal activity [20], and higher SDNN, which is influenced by both parasympathetic and sympathetic activity [21] and estimates overall HRV [14]. Bowman et al. [9] found a reduced mean HR, and increased mean RR and SDNN in dogs when initially exposed to classical music. They attributed these changes to a possible increase in vagal activity but also to the fact that the dogs spent a lot of time lying down while music was played. However, RMSSD and pNN50, both of which reflect vagal activity [11,16], were also increased, suggesting that the HRV changes resulted from increased vagal activity due to music exposure. In our study, HRV was only analysed when the dogs were lying down, but this was standardised across the four treatments, indicating that the trend in the music group were possibly driven by increased vagal activity compared to the other groups. This is supported by the trend of dogs in this group to show more behaviours indicative of relaxation. As shelters are very busy environments during the day, being able to rest more may indicate improved welfare [102]. Moreover, when physical activity is controlled, SDNN could be a good sign of increased attention [29,30]. This suggests that while dogs in the music group possibly had increased vagal activity, they could have more intently perceived the stimulus than dogs in the other groups, as music is constantly changing, opposed to DAP and lavender, which are constant. This increased attention could be reflected in some sympathetic activity, also inferred by the higher absolute LF (ms^2^) power, indicating activation of both branches of the ANS.

No significant differences between enrichment groups were found in RMSSD, pNN50 or LF/HF ratio. Köster et al. [73] did not find significant effects in RMSSD and pNN50 in dogs exposed to classical music compared to those in a silent control during a canine clinical examination practice. In that study, dogs exposed to music had higher SDNN, but lower mean RR than dogs in the control, possibly indicating that exposure to music was a novel experience rather than calming. Neither Köster et al. [73] or Bowman et al. [9] measured LF and HF bands individually, therefore it is not possible to compare directly with our results. However, they did measure the LF/HF ratio and one found it was not significant [73] while the other found it was not consistently affected by music [9].

In our previous behaviour study, dogs exposed to DAP spent more time lying down and stood on their hind legs with their front legs resting against the exit less than those in the control group. Thus dogs exposed to DAP showed more behaviours associated with increased relaxation and reduced arousal compared to the control group. The absence of any significant effects of DAP on cardiac activity is therefore surprising, but they cannot be compared with other studies, as to the authors’ knowledge, no other studies have looked into DAP effects on HRV measurements.

### 4.2. Study Limitations

One limitation for this study was the small number of dogs with baseline data and the large imbalance in dog numbers between treatments. This baseline would have been useful as an index of each subject’s autonomic state, with stressed dogs potentially having lower vagal activity before enrichment exposure [11]. The small number was in part due to the use of artefact correction, with many dogs having more than 5% artefact correction, which made them ineligible for inclusion. Having a smaller number of dogs reduced power and created imbalance across treatments, although significant differences between treatments were still apparent. Further research on the optimum level of artefact correction for studies with dogs is warranted.

Another possible limitation was the need to use an adhesive bandage to keep the heart rate monitor strap in place during the recording. Studies have shown that pressure wraps such as the ThunderShirt^®^ (Durham, NC, USA) [103] and telemetry vests [104] help to reduce heart rate and anxiety related behaviours of dogs that wear them. The bandages could have produced an anxiolytic effect and therefore reduced treatment effects. However, all the dogs (control and treatment) had the bandages applied so if there was an effect, all groups would have experienced it. Moreover, the time that the dogs had the Polar^®^ heart rate monitors attached to them might have been too long, allowing more technical issues and mechanical artefacts to occur. In the future, it would be recommended to have shorter HRV recording periods [105] to have more control over these issues. However, very short recordings would not be recommended either, as placing and adjusting the monitors might be stressful for the dogs and could influence recordings.

Motor activity can influence HRV and it can also mask emotional and health processes and produce more artefacts [16], therefore, HRV measures should be taken when stationary [11,14]. The correlation between Polar^®^ heart rate monitors and echocardiogram decreases as motor activity increases in humans [106], horses [107], pigs [108] and dogs [70]. Moreover, when the aim is to compare non-motor (psychological) components of cardiac activity, only recordings obtained during similar behavioural patterns should be used [11]. As the recordings were taken from dogs freely moving around the kennel, the only possible segments of 5 consecutive minutes in the same position were obtained when the dogs were lying down, and therefore the results reflect ANS activity only for this body posture. Despite a highly standardised protocol and obtaining the HRV measure only of stationary dogs, Essner et al. [69] found that the Polar heart rate monitor missed intervals that the echocardiogram detected and therefore some HRV results can be inaccurate. Parker et al. [107] and Marchant et al. [108] also pointed out some problems with the validity and reliability of Polar^®^ heart rate monitors and particularly when recording data in ambulatory conditions.

It is important to take into account the equipment used and its possible limitations. Following Kubios [75] advice, we used the lowest possible artefact correction level (very low threshold). However, these automatic correction levels were originally developed for human heart rate data, so there is no certainty that they can appropriately correct dog heart rate data [109].

It is possible that because HRV was only measured on the fifth day, dogs had habituated to the stimuli by then. However, based on previous behaviour observations over time [65], there was no evidence of habituation to any of the stimuli over the 5 days of exposure.

## 5. Conclusions

From the three stimuli dogs were exposed to, music produced the most changes in HRV, seemingly by activating both branches of the ANS and therefore producing significant changes in HRV parameters that reflect both parasympathetic and sympathetic activity. There were also trends for dogs in this group to have lower heart rate and consequently increased RR intervals. These results combined with the behaviour results from the first study and the behaviour results of this subset of dogs, indicate that dogs in the music group were more relaxed. There is evidence from the HRV that this was related to an increased vagal activity compared to dogs in the other groups. Shelters could consider using similar methods of music enrichment, as is it the easiest and cheapest stimulus to apply and produces both behavioural and physiological positive effects in dogs. It may help to relieve both the stress and boredom in shelter environments. As for the other stimuli, their effect might have not been strong enough to produce measurable changes in cardiac activity.

Wearable devices such as the Polar^®^ RS800CX and the Polar^®^ V800 can be useful tools to measure autonomic responses in dogs. However, many variables should be taken into account when using HRV as a physiological parameter to measure stress. These include the recording quality, the dog’s motor activity while collecting the data and artefacts [106].

## Figures and Tables

**Table 1:** Heart rate variability (HRV) parameters and their physiological origin.

Analysis Methods	Parameter	Units *	Description	Assumed Physiological Interpretation of Parameter
Time domain	Mean RR	ms	Mean time duration between successive RR intervals (two consecutive R waves of the electrocardiogram (ECG)) [9]	Increases with vagal activity and decreases with sympathetic activity [20]
	Mean HR	bpm	Mean heart rate	Increases with sympathetic activity and decreases with vagal activity [20]
	SDNN	ms	Standard deviation of RR intervals [9]	Mix of vagal and sympathetic activity [21]
	RMSSD	ms	Root mean square of differences between successive RR intervals [12]	Increases with vagal activity [11]
	pNN50	%	Percentage of successive RR interval pairs which differ by more than 50 ms [9]	Increases with vagal activity [11]
Frequency domain	LF	ms^2^ and n.u.	Low frequency band of the power spectral density analysis of the HR fluctuation (0.067–0.235 Hz) [22]	Mix of vagal and sympathetic activity [23]
	HF	ms^2^ and n.u.	High frequency band of the power spectral density analysis of the HR fluctuation (0.235–0.877 Hz) [22]	Increases with vagal activity [21]
	LF/HF		Low frequency/high frequency ratio	Mix of vagal and sympathetic activity [16]
Non-linear	SD1	ms	Standard deviation 1 of the Poincare Plot—short-term HRV [11]	Increases with vagal activity [24]
	SD2	ms	Standard deviation 2 of the Poincare Plot—long-term HRV [11]	Mix of vagal and sympathetic activity [24]

* Unit abbreviations: ms: milliseconds, bpm: beats per minute, %: percentage, ms^2^: milliseconds squared, n.u.: normalised units.

**Table 2:** HRV parameters of dogs (*n* = 34) exposed to lavender, music, dog appeasing pheromone (DAP) or a control treatment in a shelter environment, during the treatment period. For square root (√) transformed parameters, back transformed values are also reported in parentheses. Means that do not share a superscript letter are significantly different (*p <* 0.05) by Tukey’s test.

Parameters	Control	Music	Lavender	DAP	SED	F-Value (d.f. 3,29-32)	*p*-Value
Time Domain							
Mean RR (ms)	605	763	641	644	27.5	2.55	0.08
Mean HR (bpm)	103.7	80.3	95.4	96.8	3.96	2.26	0.10
SDNN (ms)	96.4	135.8	81.2	100.7	11.58	2.58	0.07
RMSSD (ms)	138	195	115	137	19.9	1.88	0.16
pNN50 (%)	52.6	69.5	50.2	52.2	6.85	1.49	0.24
Frequency Domain							
LF Power, √ (ms^2^)	46.1 ^b^ (2125)	74.8 ^a^ (5595)	39.2 ^b^ (1537)	52.7 ^ab^ (2777)	6.49	5.52	0.003
LF Power (n.u.)	35.7	36.4	35.6	39.2	5.85	0.09	0.96
HF Power, √ (ms^2^)	72.6 (5271)	103.3 (10671)	58.4 (3411)	72.6 (5271)	12.07	2.10	0.12
HF Power (n.u.)	64.1	63.3	64.2	60.5	5.85	0.09	0.96
LF/HF ratio, √	0.77 (0.59)	0.79 (0.62)	0.82 (0.67)	0.86 (0.74)	0.12	0.08	0.97
Non-linear							
SD1 (ms)	97.4	137.9	81.2	96.9	14.09	1.88	0.16
SD2 (ms)	96.1 ^ab^	130.4 ^a^	80.2 ^b^	102.3 ^ab^	10.47	3.88	0.02

Mean RR (ms; mean time duration between successive RR intervals (two consecutive R waves of the electrocardiogram (ECG)), Mean HR (bpm; mean heart rate), SDNN (ms; standard deviation of RR intervals), RMSSD (ms; root mean square of differences between successive RR interval), pNN50 (%; percentage of successive RR interval pairs that differ by more than 50 ms). LF/HF (low frequency/high frequency ratio), LF (low frequency) band (0.067–0.235 Hz) and HF (high frequency) band (0.235–0.877 Hz), both expressed in absolute values of power (ms^2^) and normalised units (n.u.). SD1 (ms; standard deviation 1 of the Poincare Plot—short-term HRV) and SD2 (ms; standard deviation 2 of the Poincare Plot—long-term HRV).

**Table 3:** The behaviour of a subset of dogs (*n* = 34) exposed to lavender, music, DAP or a control treatment, during the 3 h treatment period on 5 consecutive days. For square root (√) transformed parameters, back transformed values are also reported in parentheses. Means that do not share a superscript letter are significantly different (*p <* 0.05) by Tukey’s test.

Behaviour	Control	Music	Lavender	DAP	SED	F-Value (d.f. 3,17)	*p*-Value
Activity							
Lie down total, % of time	47.8	62.2	58.0	61.2	5.769	0.93	0.45
Lie down-head down, % of time	28.9	51.8	43.1	34.5	6.017	2.91	0.07
Lie down-head up, √ % of time	4.12 ^ab^	3.04 ^b^	3.61 ^b^	5.09 ^a^	0.437	4.79	0.01
	(17.0)	(9.24)	(13.0)	(25.9)			
Stand, % of time	34.3	30.1	31.1	26.2	4.302	0.48	0.70
Walk, √ % of time	2.69	2.12	2.27	2.26	0.248	0.67	0.58
	(7.23)	(4.49)	(5.15)	(5.10)			
Standing exit door, √ % of time	2.10 ^a^	0.82 ^b^	0.65 ^b^	0.87 ^b^	0.240	6.03	0.005
	(4.41)	(0.67)	(0.42)	(0.76)			
Sit, √ % of time	1.43	0.52	1.21	1.53	0.377	1.23	0.33
	(2.04)	(0.27)	(1.46)	(2.34)			
Vocalisation							
Vocalisation, % of time	7.90 ^a^	0.27 ^b^	1.40 ^b^	3.71 ^b^	1.427	12.2	< 0.001
Other behaviours							
Pant, √ % of time	1.07 ^a^	0.02 ^b^	0.30 ^ab^	0.33 ^ab^	0.426	4.01	0.03
	(1.14)	(0.0004)	(0.09)	(0.11)			
Body shake, √ events per hour	2.82	3.56	4.42	6.99	2.481	2.64	0.08
	(7.95)	(12.7)	(19.5)	(48.9)			
Sniff ground, √ % of time	0.29	0.19	0.22	0.44	0.144	1.25	0.32
	(0.08)	(0.04)	(0.05)	(0.19)			
Groom, √ % of time	0.41	0.34	0.54	0.82	0.271	2.80	0.07
	(0.17)	(0.12)	(0.29)	(0.67)			
Tail position and movement							
Tail low, % of time	60.2	71.6	60.2	53.8	5.353	1.26	0.32
Tail medium/high, % of time	16.3	16.1	21.1	14.4	3.828	0.26	0.86
Tail movement, % of time	11.0 ^a^	1.38 ^b^	6.82 ^ab^	6.63 ^ab^	2.164	3.37	0.04
Tail still, % of time	78.3	90.6	86.5	81.2	3.104	3.17	0.05
Location in kennel							
Front, % of time	31.0	27.2	22.6	16.6	6.623	0.66	0.59
Back, % of time	31.9	31.0	31.4	34.9	7.497	0.07	0.98
Crate, % of time	19.5	26.9	34.5	37.7	8.445	1.31	0.31
Middle, √ % of time	2.15	2.41	1.46	1.41	0.504	1.13	0.37
	(4.62)	(5.80)	(2.13)	(1.99)			

## Appendix A

**Table A1:** Dogs (*n* = 34) included in the HRV analysis.

Dog	Age (in Months)	Source	Days in Shelter at Beginning of Trial	Treatment	Heart Rate Monitor (HRM)	Number of 5-min HRV Segments
Diesel 1	15	Owner surrendered	33	Control	2	2
Sasha	37	Owner surrendered	46	Control	3	1
Diesel 2	73	Transferred from other shelter	45	Control	3	5
Koda	12	Stray	58	Control	3	4
Tyra	68	Stray	20	Control	4	5
Buffy	18	Impounded by council	30	Control	4	1
Walter	10	Impounded by council	43	Control	4	3
Chumps	28	Impounded by council	39	Control	4	2
Alf	65	Impounded by council	93	Control	2	1
Gem	50	Impounded by council	59	Lavender	4	5
Spencer	13	Owner surrendered	21	Lavender	1	1
Bob	20	Impounded by council	64	Lavender	4	1
Bronson	26	Transferred from other shelter	67	Lavender	2	2
Eugene	18	Impounded by council	22	Lavender	4	5
Chloe	18	Returned after previous adoption	22	Lavender	3	2
Diesel 3	12	Brought in by shelter ambulance	150	Lavender	3	5
Tyson	48	Impounded by council	17	Lavender	2	2
Karter	60	Stray	58	Lavender	3	1
Pumpkin	12	Impounded by council	32	Lavender	3	5
George	13	Impounded by council	37	Music	2	5
Diesel 4	25	Owner surrendered	60	Music	4	3
Rusty	44	Owner surrendered	18	Music	1	2
Oscar	60	Impounded by council	22	Music	2	5
Belle	36	Owner surrendered	60	Music	1	3
Cadbury	18	Impounded by council	24	Music	4	1
Basil	9	Impounded by council	43	DAP	4	5
Pepper	15	Owner surrendered	20	DAP	4	3
Ellie	24	Impounded by council	36	DAP	2	5
Jenny	105	Brought in by shelter ambulance	60	DAP	1	5
Mia	27	Owner surrendered	25	DAP	1	4
Missy	46	Owner surrendered	41	DAP	2	3
Lisa	6	Brought in by shelter ambulance	11	DAP	3	5
Sheba	120	Impounded by council	34	DAP	4	5
Tackle	11	Returned after previous adoption	17	DAP	4	5

## Appendix B

**Table A2:** The behaviour of shelter dogs (*n* = 60) exposed to olfactory and auditory stimuli or a control treatment for 3 h/d on 5 consecutive days [65]. For square root (√) transformed parameters, back transformed values are also reported in parentheses. Means that do not share a superscript letter are significantly different (*p <* 0.05) by Tukey’s test.

Behaviour	Control	Music	Lavender	DAP	SED	F-Value (d.f. 3,41)	*p*-Value
Activity							
Lie down total, % of time	44.4 ^b^	61.3 ^ab^	52.6 ^ab^	61.7 ^a^	4.64	3.29	0.03
Lie down-head down % of time	29.4 ^b^	49.9 ^a^	38.7 ^ab^	43.6 ^ab^	4.72	4.46	0.008
Lie down-head up, √ % of time	3.58	3.13	3.52	4.01	0.337	1.24	0.31
	(12.8)	(9.79)	(12.4)	(16.1)			
Stand, % of time	39.0	29.5	33.4	26.6	3.44	2.44	0.08
Walk, √ % of time	2.67	2.00	2.31	2.04	0.189	2.37	0.09
	(7.14)	(4.02)	(5.33)	(4.17)			
Standing exit door, √ % of time	1.67 ^a^	0.55 ^b^	0.86 ^ab^	0.51 ^b^	0.164	4.35	0.009
	(2.79)	(0.30)	(0.74)	(0.26)			
Sit, √ % of time	1.39	1.16	1.90	1.65	0.316	0.81	0.49
	(1.93)	(1.35)	(3.60)	(2.74)			
Vocalisation							
Vocalisation, √ % of time	2.42 ^a^	1.30 ^b^	1.12 ^b^	1.27 ^b^	0.291	6.90	0.001
	(5.87)	(1.70)	(1.26)	(1.61)			
Other behaviours							
Pant, √ % of time	1.30 ^a^	0.12 ^b^	0.48 ^b^	0.36 ^b^	0.267	7.26	0.001
	(1.69)	(0.01)	(0.23)	(0.13)			
Body shake, √ events per hour	0.33 ^b^	0.30 ^b^	0.42 ^b^	0.72 ^a^	0.197	6.38	0.001
	(0.11)	(0.09)	(0.17)	(0.51)			
Sniff ground, √ % of time	0.27 ^ab^	0.09 ^b^	0.25 ^ab^	0.37 ^a^	0.115	3.47	0.03
	(0.071)	(0.007)	(0.061)	(0.13)			
Groom, √ % of time	0.42	0.37	0.52	0.67	0.199	1.65	0.19
	(0.18)	(0.14)	(0.27)	(0.45)			
Tail position and movement							
Tail low, % of time	61.3	70.3	58.2	60.0	4.15	1.62	0.20
Tail medium/high, √ % of time	3.89	3.15	3.74	3.35	0.397	0.37	0.78
	(15.1)	(9.93)	(14.0)	(11.2)			
Tail movement, % of time	10.10 ^a^	5.30 ^b^	8.11 ^ab^	5.45 ^b^	1.659	3.59	0.02
Tail still, % of time	81.4	87.6	85.9	87.3	2.42	2.08	0.12
Location in kennel							
Front, √ % of time	4.90	4.70	4.23	3.51	0.501	2.10	0.12
	(24.0)	(22.1)	(17.9)	(12.3)			
Back, % of time	35.2	38.1	39.1	36.0	5.78	0.15	0.93
Crate, √ % of time	3.80	3.86	4.35	5.45	0.640	1.49	0.23
	(14.5)	(14.9)	(18.9)	(29.7)			
Middle, √ % of time	1.97	2.41	1.51	1.52	0.438	1.94	0.14
	(3.87)	(5.81)	(2.27)	(2.32)			
